# 1 km of living area: age differences in the association between neighborhood environment perception and self-rated health among Chinese people

**DOI:** 10.1186/s12889-024-18041-8

**Published:** 2024-02-23

**Authors:** Yuexuan Mu, Shu Ge, Benfeng Du

**Affiliations:** 1 School of Public health, Soochow University, Jiangsu, China; 2 School of Sociology and Population Studies, Renmin University of China, Beijing, China; 3 Special expert of Henan Provincial Government, Henan, China

**Keywords:** Neighborhood environment perception, Built environment, Social environment, Age difference, Self-rated health

## Abstract

**Objectives:**

This study aimed to explore the age differences in the relationship between neighborhood environment perception and self-rated health among Chinese people.

**Study design:**

This is cross-sectional study.

**Methods:**

The participants were 2,631 residents aged 18 and above from 2021 Chinese General Social Survey (CGSS). Self-rated health was reported by residents. Neighborhood environment was measured by respondents’ subjective perception of 1 km living area. Ordered logit regression models were used to examine the relationship between neighborhood environment perception and self-rated health.

**Results:**

In summary, 42.08% were classified as young adults, and 57.92% were classified as middle-aged and older adults. Young adults with higher perception of neighborhood social environment were more likely to perceive good health. Neighborhood built environment was significantly associated with self-rated health among middle-aged and older adults.

**Conclusion:**

The neighborhood environment is an important predictor of the health of its residents. Neighborhood environmental modifications should be tailored to meet the needs of different age groups, promoting health equity.

## Introduction

Self-rated health is a commonly used health indicator that assesses an individual’s overall health status by combining subjective and objective health information. This assessment is consistent with the “objective” health assessment conducted by physicians and provides insight into an individual’s quality of life [1]. The concept has been utilized in numerous related studies and holds scientific validity [2, 3].

In 2016, China issued and implemented the “Healthy China 2030” plan, which emphasizes that the improvement of national health should be accomplished by connecting society as a whole and achieving the integration of “health in all policies”, instead of relying solely on the healthcare system. This means that policymakers are focusing on the “upstream” social influences on health and exploring in-depth the social and environmental factors that affect health. In 2019, The State Council’s General Office has released a plan on the implementation and evaluation of the “Healthy China” initiative. This plan stated the importance of focusing on the creation of a healthy environment in the community and strengthening the role neighborhood in health promotion. Strengthening collaboration with multiple departments to create sustainable and supportive environments has been recognized as an important strategy for enhancing health. In the Sustainable Development Goals issued by the United Nations, it is also stated that the construction of sustainable cities and communities ensures healthy lives and promotes well-being [4]. The World Health Organization reiterated the health-promoting role of urban planning in the Shanghai Declaration [5]. In summary, the neighborhood has become a crucial aspect of daily life for residents. Strengthening the neighborhood environment to enhance the quality of life and promote better health for residents has become a pressing social concern.

Stress Process Model was introduced by Pearlin in 1981 and it presents the process of stress, including exposure to stressors, personal coping resources, and mental health outcomes [6, 7]. The model suggests that stressors can directly or indirectly influence individuals’ life satisfaction, subjective and psychological well-being [8, 9]. Based on the stress process model, individuals are exposed to their neighborhood environment, and the resources available in the neighborhood can act as environmental stressors [7, 10]. Several studies have applied the stress process model to explain the relationship between neighborhood environment and health [11–13]. The neighborhood environment generally includes the built and social environments [14, 15]. The built environment typically includes a variety of street-level features, including public space design, sidewalks, and intersections [16]. It also includes community-level features, such as land use characteristics, building density, and the accessibility of green space [17]. The social environment is characterized by the quality of relationships within a neighborhood, which can be assessed through social trust, connectivity, and social cohesion perceived by its residents [18].

Neighborhood environmental perceptions refer to individuals’ subjective feelings and psychological judgments regarding their surrounding environment and any changes that occur within it [19]. Yu et al. applied multivariate regression models to study Hong Kong older adults [20]. They found that the neighborhood environment perception had a significant effect on the self-rated health. Lu et al. also found the significant association between neighborhood environment perception and self-rated health among older adults [21]. Besides, they also identified the moderation role of self-rated health in the relationship between neighborhood environment perception and depressive symptoms with structural equation modeling. Although Lyu et al. found that the built environment was only associated with self-rated health in older people, and this association did not exist in young and middle-aged people, they neglected to consider the social environment [22]. Additionally, they only included a sample from one province in China.

Most existing studies have focused on middle-aged and older groups, but neighborhood environments include residents of all ages. If we prioritize only the renovation and construction of neighborhood environments for older residents, we run the risk of neglecting the healthy development of younger residents. This could ultimately threaten social stability. This study differentiates between age groups and examines the impact of neighborhood environment perception on their self-rated health, providing more precise empirical evidence to enhance neighborhood environments and improve health outcomes.

**Fig. 1:**
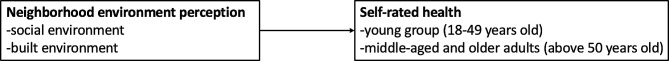
Research framework

Furthermore, the survey results may be biased because residents may have a limited understanding of the neighborhood’s scope. Often, their perception of the neighborhood is narrower than the boundaries officially defined. This can lead to an underestimation of the actual health effects of the neighborhood environment, which could potentially affect the implementation of policies. This study employed a 1 km radius as the scope of the neighborhood environment to improve the understanding of the true health effects of the neighborhood environment (Fig. 1). This will enable the development of a more precise plan to improve the neighborhood environment.

## Data and method

### Study participants

This study used data from Chinese General Social Survey (CGSS). CGSS is the earliest national representative continuous survey project run by Renmin university of China. It is a continuous cross-sectional survey which collect data on multiple levels of Chinese society, communities, households, and individuals. The survey covers 28 provinces in China, which has a good representation. CGSS launched in 2003 and 2021 is the 14th annual survey. We selected the latest version of data in 2021. 8148 samples were collected in 2021. However, questions related to health and neighborhood were only included in a specific theme module. CGSS randomly selected a third of the survey respondents to answer. 2717 residents were interviewed in this module. Based on this initial sample, we further removed missing values in the environment variable responses, totaling 86. The final sample size was 2631.

### Measurements

#### Self-rated health

Individuals respond to the question, “What is your current state of health?”. Respondents rated their health condition on a five-point scale (Excellent; very good; good; average; poor). Participants’ responses were recoded as the categorical variable.

#### Neighborhood environment

Neighborhood environment is defined as a 1 km living area (approximately 15 min on foot). Investigators would remind respondents about the specific range of 1 km. Investigators would ask for a 15-minute radius of residence if respondents do not have a clear sense of location. The study is specifically about respondents’ subjective perception of the neighborhood environment, including built environment and social environment.

The neighborhood built environment include outdoor space, household goods, and public facilities. Specifically, respondents were asked to what extent they agreed or disagreed with the following three questions: (1) “The environment is good for outdoor activities, such as jogging or walking”; (2) “There are plenty of fresh fruits and/or vegetables available in the living area”; and (3) “There are adequate public facilities in the neighborhood environment, such as parks, community centers, libraries.” Each question had five options, ranging from 1 = strongly disagree to 5 = strongly agree.

For the measurement of neighborhood social environment, respondents were asked to what extent they agreed or disagreed with the following three items: (1) “Neighbors care about each other,” (2) “Neighbors are safe,” and (3) “Neighbors are willing to help when I am in need.” Each question had five response options, ranging from 1 = Strongly disagree to 5 = Strongly agree.

#### Age groups

This study divided respondents into two age groups (1 = 18–49 years old; 2 = above 50 years old). People over the age of 50 are often classified as middle-aged and older group [23]. In China, individuals begin to gradually transition into full-time retirement at 50 years old [24]. Previous studies have showed that middle-aged and older adults tend to primarily stay in their local neighborhoods, in contrast to younger people who may be exposed to a variety of environments, including work and school [25, 26].Therefore, the impact of the neighborhood environment on residents needs to consider this difference.

#### Covariates

Neighborhood environment is related with sociodemographic characteristics, and such variables have also been predicted to influence self-rated health. Thus, we included sex (1 = male; 2 = female), place of residence (1 = urban; 2 = rural), years of education, marital status (0 = unmarried; 1 = married), and household income as potential confounding factors.

### Statistical analysis

First, we conducted a descriptive analysis to understand the basic distribution of each variable. Categorical variables were expressed as the number and percentage and continuous variables were reported as mean and standard deviation. Besides, ANOVA test was used to analyze the difference between the means of the two groups (young adults vs. middle-aged and older adults). Third, we conducted a correlation analysis to measure the strength of the relationship between neighborhood environment and self-rated health.

Finally, ordered logit regression models were performed to examine the relationship between neighborhood environment and self-rated health. We used the “perceived poor health” category as the reference. There were three models in current study: model 1 was adjusted for sociodemographic indicators (age, gender, years of education, marital status, place of residence and income); model 2 and model 3 were based on the subgroup analysis. The simple correlation coefficient test was performed to examine the presence of severe multicollinearity and all coefficients were all less than 0.8. Software Stata 15.0 was used in all statistical analyses. *P* < 0.05 was statistically significant.

## Results

Table 1 shows the basic characteristics of residents by age groups. The study included 2631 samples, ranging in age from 18 to 99 years old, with an average age of 51.9 years. The total sample included 1434 females and 1197 males, with an average education level of 9 years, which is equivalent to a junior high school education. About 60% of residents live in rural areas, 71% are married, and over 90% believe that their family’s annual income level is at a medium or lower level. The proportion of individuals who self-rated their health as “fair” was the highest.

**Table 1:** Basic information about individuals in 2021

Categorical variable	Total sample	Age group 1 ($$ <$$50)	Age group 2 ($$ \ge $$50)	$$ {\chi }^{2}$$	*P*
	N (%)	N (%)	N (%)		
Self-rated health				258.93	$$ <$$0.001
Poor	297 (11.29)	42 (3.79)	255 (16.73)		
Average	943 (35.84)	291 (26.29)	652 (42.78)		
Good	453 (17.22)	226 (20.42)	227 (14.90)		
Very good	618 (23.49)	354 (31.98)	264 (17.32)		
Excellent	320 (12.16)	194 (17.52)	126 (8.27)		
Gender				6.30	0.012
male	1197 (45.50)	472 (42.64)	725 (47.57)		
female	1434 (54.50)	635 (57.36)	799 (52.43)		
Place of residence				-4.29	0.038
urban	1063 (40.40)	473 (42.73)	590 (38.71)		
rural	1568 (59.60)	634 (57.27)	934 (61.29)		
Marital status				37.74	$$ <$$0.001
unmarried	746 (28.35)	384 (34.69)	362 (23.75)		
married	1885 (71.65)	723 (65.31)	1162 (76.25)		
Household income				28.19	$$ <$$0.001
lower than average	1062 (40.36)	382 (34.51)	680 (44.62)		
average	1358 (51.62)	634 (57.27)	724 (47.51)		
higher than average	211 (8.02)	91 (8.22)	120 (7.87)		
**Continuous variable**	$\stackrel{-}{x}\left(sd\right)$	$\stackrel{-}{x}\left(sd\right)$	$\stackrel{-}{x}\left(sd\right)$		
Neighborhood environment					
Built environment	11.32 (2.36)	11.51 (2.16)	11.19 (2.48)	3.20	$$ <$$0.001
Social environment	12.68 (1.75)	12.42 (1.71)	12.84 (1.76)	-5.40	$$ <$$0.001
Years of education	9.28 (4.73)	11.82 (3.99)	7.42 (4.34)	26.48	$$ <$$0.001
$$ N$$	2631	1107	1524		

In summary, 1107 (42.08%) were classified as young adults, and 1524 (57.92%) were classified as middle-aged and older adults. According to the results of the chi-square test, young adults were more likely to perceive good health, living in urban area, having higher household income and higher satisfaction with the built environment than older individuals. Besides, Residents over 50 years old report higher levels of satisfaction with the social environment compared to those under 50.

Table 2 showed the results of the correlation analysis. In the total sample, self-rated health is significantly positively correlated with built environment and social environment, and built environment is also significantly positively correlated with social environment. After conducting correlation analysis by age groups, self-rated health is still significantly positively correlated with built environment and social environment.

**Table 2:** Correlation analysis among neighborhood environment and self-rated health

	Total sample			Age group 1 ($$ <$$50)			Age group 2 ($$ \ge $$50)		
	1	2	3	1	2	3	1	2	3
Self-rated health	$$ -$$			$$ -$$			$$ -$$		
Built environment	0.140^***^	$$ -$$		0.084^*^	$$ -$$		0.151^***^	$$ -$$	
Social environment	0.056^*^	0.268^***^	$$ -$$	0.107^**^	0.264^***^	$$ -$$	0.089^**^	0.291^***^	$$ -$$

Notes: *P< 0.05; **P< 0.01; ***P<0.001

The association between neighborhood environment and self-rated health was presented in Table 3. In total sample, built environment and social environment were associated with self-rated health. People with higher perception of neighborhood environment were more likely to perceive good health in model (1) In subgroup analysis, social environment was associated with self-rated health. Young adults with higher perception of social environment were more likely to perceive good health in model (2) Built environment was associated with self-rated health. Middle-aged and old adults with higher perception of built environment were more likely to perceive good health in model 3.

**Table 3:** The influence of neighborhood environment on self-rated health

	Total sample		Age group 1 ($$ <$$50)		Age group 2 ($$ \ge $$50)	
	Coef. ($$ SE$$)	$$ OR$$	Coef. ($$ SE$$)	$$ OR$$	Coef. ($$ SE$$)	$$ OR$$
Built environment	0.063^**^ (0.020)	1.065	0.003 (0.035)	0.997	0.085^***^ (0.025)	1.088
Social environment	0.075^**^ (0.026)	1.078	0.124^**^ (0.042)	1.132	0.036 (0.034)	1.036
Control variables	Yes	Yes	Yes	Yes	Yes	Yes

Notes: *P< 0.05; ** *P*< 0.01; ***P< 0.001

## Discussion

To our best knowledge, this is the first study to compare age difference in the neighborhood environment perception and self-rated health with national representative data from China. This study comprehensively considered the needs of both young adults and middle-aged and old adults in the neighborhood environment as the residents in the community are not limited to a single age group. The results aimed to explore measures to improve the self-rated health of individuals in different age groups from the perspective of neighborhood environment.

Firstly, this study found the significant association between neighborhood environment perception and self-rated health in total sample. This positive result can be explained by person-environment fit theory [27]. According to this theory, the interaction between people and their environment is dynamic. Residents feel more comfortable in their daily lives when they are fit with their neighborhood environment. Combined with the understanding from theories and empirical evidences, supportive neighborhood environments promote residents’ healthy behaviors and social trust such as less sedentary behavior, more social activities, and the sense of security [28, 29]. At this time, people are more likely to have higher self-rated health because they achieve a match between individual competence and environmental press.

Secondly, this study found the age difference in neighborhood environment perception and self-rated health. There were significant differences in psychological demand and physical function throughout the lifespan [30, 31]. Besides, middle-aged and old adults have more flexible time than young people because of retirement and aging so that middle-aged and old adults may spend more time in the neighborhood. Thus, residents from difference age groups have difference priority in neighborhood environment perception.

According to the research results, the young group aged 18–49 attaches more importance to the neighborhood social environment. In previous studies, the neighborhood social environment has been found to encompass factors such as public participation, neighborly relations, residents’ sense of belonging and identity [32]. In this study, the measurement of neighborhood social environment included neighborhood trust and security. It indicated that neighborhood social environment in this study was prone to residents’ sense of belonging and security, rather than social activities participation. Previous studies showed that young people are much more pessimistic than older adults about certain social trust issues [33]. Furthermore, rapid economic and social changes, coupled with stressful work and life pressures, can make young people more susceptible to anxiety and despair [34]. From a theoretical perspective, the collective efficacy theory can help us understand this association [35]. Based on the theory, risky and problematic behaviors may decline due to collective social control. A disadvantaged social environment can increase the vulnerability and fear of young residents, resulting in heightened psychological pressure [36]. However, there was no significant association between neighborhood social environment and self-rated health among middle-aged and older adults. According to the study by Simran, the impact of the neighborhood may decrease as residents age [37]. To interpret this, middle-aged and older adults may rely more on close networks, such as family members, and less on perceived support from neighbors. This may result in insignificant impact of neighborhood social cohesion on middle-aged and older adults’ self-rated health [38, 39].

This study found that the built environment is significantly associated with self-rated health among middle-aged and older adults. In this study, the assessment of the built environment includes outdoor spaces and daily living facilities, which reflect the level of convenience in community living. Middle-aged and older individuals have more free time than younger individuals. Moreover, as individuals age, middle-aged and older adults experience a gradual decline in physical functioning, which diminishes their ability to travel long distances [40]. As a result, unlike younger people who have more social options and are in better physical shape, middle-aged and older people spend more time in the community. The outdoor space and park provide middle-aged and older people with more opportunities to exercise, thereby improving their self-rated health [41, 42]. Besides, young people may be more likely to choose online apps to achieve the convenience of daily life. Because of the digital divide, the accessibility and convenience of surrounding facilities, such as supermarkets and convenience stores, can directly impact the life satisfaction of middle-aged and older adults and further influence their self-rated health [43]. To explain the insignificant association between the built environment and self-rated health among young individuals, it is important to consider that they spend less leisure time in their neighborhood due to work and other social activities conducted outside. As a result, the convenience of the built environment may not have a profound influence on their daily life.

The research still has certain limitations. Firstly, the study utilized cross-sectional data, which cannot establish a causal relationship between the neighborhood environment and self-rated health. Future research could utilize panel data to analyze both the short-term and long-term effects of exposure to neighborhood environments on self-rated health. Secondly, the study employed quantitative analysis. Future research can combine interviews to further explore the impact mechanism of neighborhood environment on self-rated health among different groups. Thirdly, this study did not provide a detailed explanation of the spatial differences. Therefore, future studies may incorporate an examination of spatial differences.

This article has several policy implications. To improve the health conditions of residents, focusing on the neighborhood environment is a more effective starting point. When health and other related departments consider the reconstruction and improvement of neighborhood environments, it is necessary to pay more attention to the age composition of different communities. For communities with a higher proportion of younger residents, it is necessary to enhance security measures and foster a sense of belonging. Specifically, more patrols can be added and surveillance can be installed around the neighborhood. At the same time, community staff can organize neighborhood activities to encourage young people to meet their neighbors and strengthen their sense of belonging. For communities with a higher proportion of middle-aged and older residents, the government needs to improve community accessibility. For example, it should expand the number of exercise areas for middle-aged and older people and increase the availability of exercise equipment that is suitable for their needs. Supermarkets and pharmacies should be opened near the community to facilitate the daily needs of middle-aged and older people.

## Conclusion

This study examined the relationship between neighborhood environment perception and self-rated health in two different groups: young adults, middle-aged and older adults. The data used for this analysis was obtained from the Chinese General Social Survey (CGSS). We found that the social environment significantly associated with self-rated health among young adults, while built environment significantly associated with self-rated health among middle-aged and older adults. The results indicated that further attention should be given to the construction of a healthy neighborhood environment. The age composition of every community needs to be noticed, and effective reform measures can be implemented for different age groups.

## Data Availability

On reasonable request, these data may be made available from the corresponding author.
